# The Role of Diet in the Management of Irritable Bowel Syndrome: A Comprehensive Review

**DOI:** 10.7759/cureus.54244

**Published:** 2024-02-15

**Authors:** Maleesha Jayasinghe, Vinuri Karunanayake, Ali Mohtashim, Dilushini Caldera, Piyalka Mendis, Omesh Prathiraja, Fatemeh Rashidi, John A Damianos

**Affiliations:** 1 Medicine, Nanjing Medical University, Nanjing, CHN; 2 Internal Medicine, Nanjing Medical University, Nanjing, CHN; 3 Medicine, Allama Iqbal Medical College, Lahore, PAK; 4 Medicine, 37 Military Hospital, Accra, GHA; 5 Medicine and Surgery, Nanjing Medical University, Nanjing, CHN; 6 Gastroenterology and Hepatology, Mayo Clinic, Rochester, USA

**Keywords:** irritable bowel syndrome, treatment of ibs, diagnosis of ibs, mediterranean diet (md), gluten-free diet, low fodmap, fodmap diet, ibs (irritable bowel syndrome), ibs treatment, ibs

## Abstract

Irritable bowel syndrome (IBS) is a highly prevalent gastrointestinal disorder that has a significant impact on the general population. The suboptimal medical treatments available for IBS contribute to its large economic burden. The pathophysiology of IBS is complex, and treatments often focus on managing specific symptoms. Many individuals with IBS associate their symptoms with specific food intake, leading to increased scientific research on the role of diet in managing IBS. Dietary management has become a crucial aspect of IBS treatment, with initial recommendations focusing on adopting a healthy eating pattern and lifestyle. This comprehensive review aims to synthesise the current literature on the impact of diet on IBS, exploring various dietary approaches to managing IBS, including the low fermentable oligosaccharides, disaccharides, monosaccharides, and polyols (FODMAP) diet, gluten-free diet, Mediterranean diet, and tritordeum-based diet. It presents evidence from both experimental and observational studies and summarises the underlying dietary triggers in IBS, including gut microbiota dysbiosis, visceral hypersensitivity, and immune activation. In addition, it explores the efficacy and limitations of the key diet and lifestyle recommendations provided by dietary guidelines and scientific literature, highlighting the importance of individualised dietary strategies tailored to the unique needs of different types of IBS patients. By elucidating the complex interplay between diet and IBS pathophysiology, this review provides valuable insights into optimising dietary management approaches for improving symptom control and enhancing the quality of life for individuals with IBS.

## Introduction and background

Irritable bowel syndrome (IBS) is the most commonly diagnosed gastrointestinal (GI) disorder. It is defined as a disorder of gut-brain interaction (formerly termed “functional”) characterized by chronic, recurrent abdominal pain associated with a change in bowel habits, and often accompanied by bloating and depending on the subtype, urgency and/or a sensation of incomplete evacuation [1,2]. Patients suffering from this disease often have more healthcare contact for hospitalization, diagnosis, and treatment and consume more medication than people who do not have this condition, thus severely affecting their work productivity and quality of life [3]. Although a large proportion of patients do not seek medical assistance, IBS is still responsible for 20% to 50% of gastroenterology clinic visits [4,5]. The disease has a global prevalence of 5% to 10% with a female predominance with most cases developing in early childhood, although prevalence is highest in early adulthood [1,6].

The exact pathophysiology of the disease is incompletely elucidated and is multifactorial. Genetic factors, diet, abnormalities in the gut microbiome, infections, and psychological factors are all contributors to the modulation of the bi-directional gut-brain axis which appears to underlie IBS pathogenesis [3,7]. 

Genetics

Several studies have found that genetic polymorphisms in the promotor region of the solute carrier family 6 member 4, which encodes the serotonin reuptake transporter may be associated with IBS [8]. Genome-wide association studies have found that alterations in genes encoding sucrose-isomaltase or sodium channel protein type 5 subunit alpha (a voltage-gated sodium channel) affect smooth muscle function and mechanical sensitivity, explaining IBS-type symptoms in a subset of patients [6]. Additionally, variations in a locus on chromosome 9 (9q31.2 locus) are found to be associated with IBS in women and familial dysautonomia which might explain autonomic dysfunction in IBS [8]. Also, hereditary alpha tryptasemia (HaT) has been associated with IBS-type symptoms and dysautonomia. Konnikova et al. found that HaT causes subclinical intestinal inflammation in patients with IBS. The inflammation was attributed to an increase in mast cell numbers, higher intestinal epithelial cell pyroptosis, and immunoglobulin (Ig)G reactive to GI-related proteins in the gut and the peripheral blood [9]. Findings from twin studies demonstrate that there is an increased concordance of IBS in monozygotic twins than dizygotic twins [10]. However, having a mother with IBS is a stronger risk factor, suggesting that environmental factors such as learned behaviour play a more important role than genetic factors [6]. The role of gene mutations in the pathophysiology of IBS remains unclear despite several studies.

Diet

Studies consistently show that a diet rich in fermentable oligosaccharides, disaccharides, monosaccharides, and polyols (FODMAPs) increases intestinal water content and colonic volume causing osmotic diarrhea, and as these sugars are poorly absorbed, fermentation results in gas production and luminal distension [6]. This is particularly true in patients with visceral hypersensitivity, such as in IBS. Reducing the amount of FODMAPs provides symptomatic relief in many patients. However, the symptomatic benefit seen in IBS patients after following a FODMAP diet varies from 50% to 86% therefore more randomized controlled trials (RCT) should be done to prove that the FODMAP diet is effective than other diet plans [8]. Studies have also shown that gluten-free diets are beneficial for IBS patients. The benefits of Gluten-free diets are attributed to fructans which is a FODMAP, therefore IBS patients following a gluten-free diet will incorporate the benefits of a low FODMAP diet [6]. Gluten-containing diets result in an increased expression of immune markers, toll-like receptor 2, and regulated T-regulatory cell marker, forkhead box P3 protein. It also affects the expression of small bowel barrier proteins and increases the mucosal permeability contributing to the pathogenesis of IBS [11].

Gut microbiome

The intestinal microbiome consists of millions of commensal bacteria, viruses, fungi, and archaea commensal bacteria. The composition of the microbiota can promote a variety of health and disease states. The fecal microbiota of patients with IBS (microbiome ‘signature’) is considerably different from that of healthy individuals. The dysbiotic findings seen in IBS patients include reduced microbial diversity, the presence of pathogenic Streptococcus species and Clostridiae species, and a reduced abundance of beneficial Bifidobacteria [3,8]. IBS is strongly associated with small intestinal bacterial overgrowth (SIBO) (odds ratio: 3.45 to 4.7), with some estimates that up to 60% of IBS features are due to SIBO [3,12]. A questionnaire-based study that assessed pain in IBS patients found that the amount of Bifidobacteria was more than five-fold lower in patients who experienced pain than those without pain [3]. Additionally, several studies have identified low-grade inflammation in IBS as a cause of visceral hypersensitivity, epithelial and neuromuscular dysfunction, and altered motility [3,6]. Antibiotics are also responsible for altering the gut microbiota and the development of IBS [6,8].

Infection

Enteric infection is one of the strongest risk factors for IBS, a phenomenon termed post-infectious IBS. A study reported that one-fourth of the patients who developed gastroenteritis six months before complained of persistent altered bowel movements, with a 7.14% chance of developing IBS [8]. Changes in the microbiota and intestinal permeability are brought on by gastrointestinal infections. These modifications may encourage immune cell activity, such as that of T- T-cells and mast cells leading to the release of cytokines, which alters motor, sensory, and secretory activities of the GI tract [3].

Psychological factors

Psychological comorbidities such as stress, anxiety, or depression are often associated with IBS and may contribute to symptoms. The bi-directional gut-brain axis is important in the pathophysiology of IBS. The psychological symptoms can alter the gut physiology and sensation triggering IBS symptoms. Similarly, the changes in the gut can negatively affect an individual’s mental well-being. Studies have reported anxiety and depression to be strong predictors of IBS at one year of follow-up [8].

**Figure 1:**
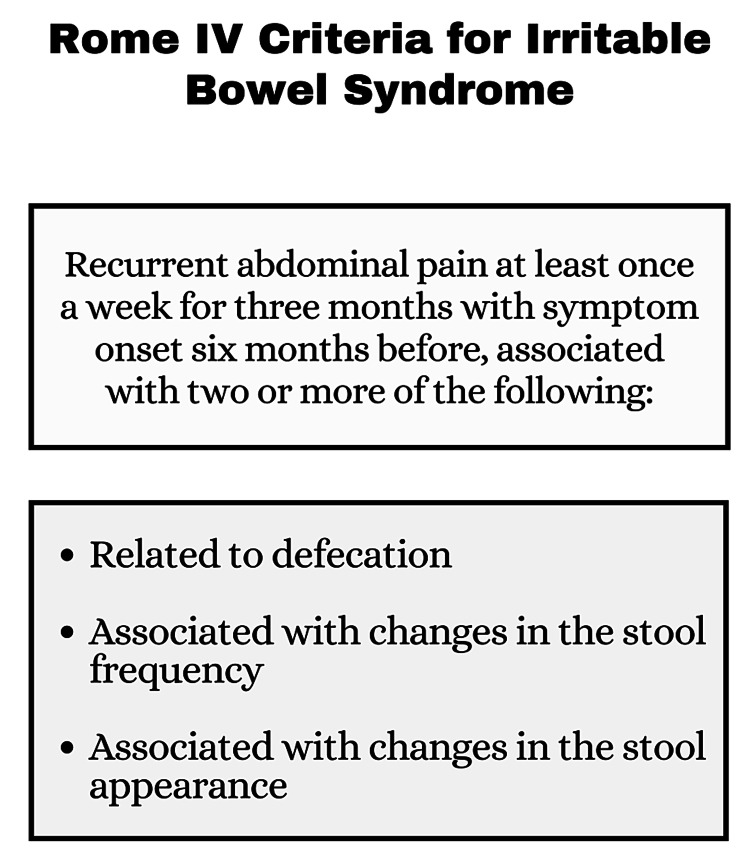
Rome IV Criteria Image Credits: Dr. Vinuri Karunanayake

Diagnosis of IBS is based primarily on the patient’s symptoms and basic investigations to rule out certain disorders resembling IBS [7]. The latest symptom-based diagnostic criteria for IBS is the Rome IV criteria (Figure 1).

Based on the Rome IV criteria, IBS is subtyped into four categories according to the stool pattern described by the Bristol Stool Scale (Table 1) [13,14]. 

**Table 1:** Subtype classification of irritable bowel syndrome

Irritable bowel syndrome (IBS) subtype classification	
IBS subtype	Stool appearance
Constipation predominant IBS (IBS-C)	>25% hard stools, <25% loose stools
Diarrhea predominant IBS (IBS-D)	>25% loose stools, <25% hard stools
Mixed IBS (IBS-M)	>25% loose stools, >25% hard stools
Unclassified IBS (IBS-U)	<25% loose stools, <25% hard stools

**Figure 2:**
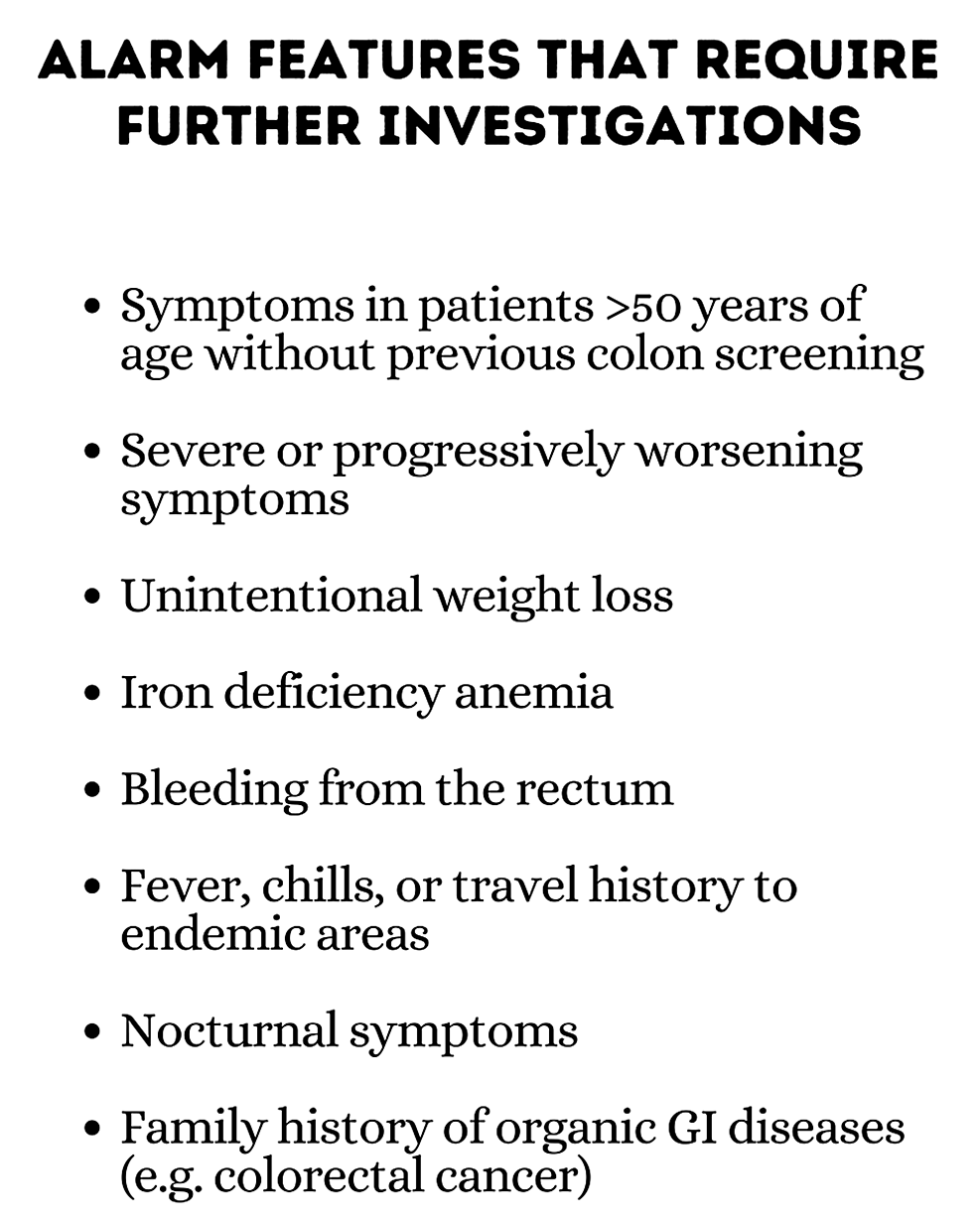
Alarm features GI: Gastrointestinal Image Credits: Dr. Vinuri Karunanayake

Careful history-taking and physical examination are important in excluding other diseases, with special attention given to alarm symptoms (Figure 2). 

Colonoscopy should be pursued in patients presenting with alarm symptoms and with a family history of early-onset colorectal cancer. For other cases, a full blood count, C-reactive protein (CRP), and fecal calprotectin levels should be tested to rule out anemia or inflammation. Enteric pathogen panel, thyroid function tests, and celiac disease serology are recommended for patients presenting with diarrhea. In certain cases, breath tests for lactose malabsorption and scintigraphic or laboratory evaluation for bile acid malabsorption may be done [15]. Additionally, potential novel biomarkers have been identified in IBS as a result of studies concerning the role of gastroenteritis and microbiome dysfunction in the pathogenesis of IBS. These biomarkers include cytolethal distending toxin B (CdtB), produced by gastroenteritis-causing bacteria, and vinculin, which was found to cross-react with CdtB antibodies. Both these biomarkers were found to be elevated in Irritable Bowel Syndrome with Diarrhea (IBS-D) patients when compared with Irritable Bowel Syndrome (IBD) patients, celiac disease patients, and healthy individuals, enabling the ability to rule in IBS-D from other patients [16]. Another study found that both anti-CdtB and anti-vinculin biomarkers were elevated in subgroups, of irritable bowel syndrome with constipation (IBS-C) and IBS-D, while only anti-vinculin biomarkers were elevated in irritable bowel syndrome mixed type (IBS-M) subgroups [17]. 

Treatment of IBS should include a thorough explanation of the disease and the gut-brain axis. In a randomized controlled study, well-structured patient education on IBS showed a significant improvement in the symptoms [6]. Symptom management of IBS includes non-pharmacological and pharmacological options. 

Lifestyle changes, dietary modifications, physical activity, and stress reduction are the most effective non-pharmacological methods of treatment [15]. A randomized controlled study reported that exercise under the guidance of a physiotherapist considerably improved the symptoms of IBS patients when compared to a control group that had no exercise [6]. Also, relaxation training, gut-directed hypnotherapy, and cognitive behavioral therapy (all of which target the psychosocial factors contributing to IBS) may be beneficial for patients [15]. Microbiome-targeted therapies such as prebiotics, probiotics, synbiotics, antibiotics, and fecal microbiota transplantation have been found to play a role in the management of symptoms in some IBS patients. They work by restoring eubiosis, reducing inflammation, regulating gut motility, modulating bile acid metabolism, and changing the intestinal bacterial profile [11,15,18]. 

Pharmacological treatment depends on the subtype of IBS. IBS-D type can be treated with peripheral opioid receptor agonists such as loperamide and eluxadoline, bile acid sequestrants (e.g. cholestyramine, colestipol), tricyclic antidepressants, serotonin 5-hydroxytryptamine 3 (5-HT3) receptor antagonist such as alosetron and antibiotics like rifaximin. IBS-C therapies include osmotic laxatives (such as polyethylene glycol), soluble fiber, prostaglandin derivatives, selective serotonin reuptake inhibitors, serotonin receptor 5-hydroxytryptamine 4 (5-HT4) agonists, guanylate cyclase-C agonist (GC-C agonist) and gastrointestinal sodium/hydrogen exchanger NHE3. Out of these, osmotic laxatives and bulking agents are considered first-line therapies in IBS-C patients. Prostaglandin derivatives such as lubiprostone increase intestinal lumen water content, and serotonin receptor 5-HT4 agonists such as tegaserod and prucalopride act as prokinetic agents, thereby benefiting IBS-C patients. GC-C agonists such as plecanatide and linaclotide are also FDA-approved for IBS-C. They increase small intestinal fluid secretion. Gastrointestinal sodium/hydrogen exchanger NHE3 such as tenapanor reduces intestinal absorption of sodium and phosphate thereby increasing luminal fluid [10,15,19,20]. Compared to the IBS-C and IBS-D subtypes, the treatment of IBS-M is a therapeutic challenge as there are no specific drugs to treat this type. Periods of constipation are treated with bulking agents and osmotic laxatives, while periods of diarrhea are treated with anti-diarrheal drugs. It is important to titrate doses according to the stool consistency and frequency to avoid worsening symptoms [15]. Global symptom relief and reduced abdominal pain can be achieved by the use of antispasmodics, peppermint oil, antidepressants, and antibiotics. Antidepressants are used for their neuromodulatory effects, having the ability to increase endorphin release and activate descending inhibitory pain pathways; therefore they can be used in cases of chronic abdominal pain [11,15].

The British Society of Gastroenterology guidelines suggest that dietary advice should be the first-line approach in the treatment of IBS. Elimination diets such as low FODMAP diets, gluten-free diets, and traditional dietary advice to eat small regular meals, avoid trigger foods, and cut back on alcohol and caffeine have been shown to greatly improve symptoms of IBS [6,15]. Diet has a significant effect on the health-related quality of life of IBS patients as well as on the management of IBS symptoms. According to some studies, some foods may be linked to histamine release and gut inflammation, which may worsen the gastrointestinal system-related symptoms of IBS [21]. The pathophysiology of IBS is still incompletely understood, even though, recent research has established that it is primarily caused by disordered communication between the gut and the brain, resulting in changes in gut motility, visceral hypersensitivity, and altered CNS processing [6]. More research is required to understand microbiome variations among IBS subtypes, as well as treatment methods that may benefit each subtype in terms of dietary interventions. Another area worth researching is metabolomics, which is the comprehensive study of small metabolic products produced by cells and tissues at a particular time [22]. This will shed more light on the complex interplay between metabolites in the gut microbiota. To advance the creation of individualized treatment choices for patients with IBS, it will be critical to design clinical trials to examine the impact of different dietary and environmental factors on these patients.

Due to the complexity of IBS and how symptoms relate to food, it is essential to use a multidisciplinary strategy that includes the patient, a healthcare provider, and a registered dietician, if one is accessible, to start customized dietary interventions as the first management step. To comprehend the connections between dietary intake and patients' IBS-specific symptoms, it is also crucial to obtain a detailed dietary history, including the types of food they consume and their eating habits. As eating disorders are prevalent and frequently overlooked in gastrointestinal conditions (particularly avoidant-restrictive food intake disorder), routine screening is essential when obtaining a dietary history. Collaboration with gastrointestinal psychology can help identify and treat disordered eating patterns. A food diary is a helpful tool to determine the relationship between dietary patterns and IBS symptoms [23,24].

Traditional dietary advice (TDA), a gluten-free diet (GFD), low FODMAP, and the Mediterranean diet have been the center of research into dietary therapies for IBS (LFD). The current recommendations for the use of dietary therapies in IBS differ widely across the globe. For example, British guidelines recommend using TDA followed by the LFD, to manage IBS, whereas American and Canadian guidelines only recommend using the LFD [24]. This could be due to differences in dietary habits between countries. For instance, typical Asian diets contain more fiber than the majority of Western diets. As a result, the approach for dietary habit modification and diet education in Asian patients with IBS may vary from that in Western patients [25]. It is important to recognize the impact that diet can have on managing symptoms of IBS and the need for personalized treatment options based on individual dietary habits and needs. It is also important to note the potential differences in dietary recommendations based on cultural and ethnic backgrounds. Further research in this area, including in pediatric populations, will be valuable in developing effective dietary interventions for those with IBS.

## Review

Method

The databases searched include PubMed, PubMed Central (PMC), ResearchGate, and Google Scholar. Relevant keywords and Medical Subject Heading (MeSH) terms were used to identify all relevant articles on the role of diet in the management of IBS. Both MeSH terms "IBS" and "Diet" were included in the search. Articles from 2015 to 2023 were included, encompassing reviews, animal studies, observational studies, and clinical trials. Only studies with accessible full-text articles and published in English were considered for inclusion.

Nutrition-gut-brain interaction

The gut-brain axis is a bi-directional pathway connecting the gastrointestinal system with the central nervous system via neural, hormonal, and immunological signaling. The gastrointestinal system houses a large community of microbes personalized to an individual in the small and large intestines [26,27]. Important physiologic barriers are the blood-brain barrier and the intestinal barrier, which are dynamic. In a healthy person, these barriers are functional [28].

Internal physiological conditions are sensed through interoception; however, gut microbes and their metabolites also contribute considerably [28]. The reciprocal associations among the brain (via the autonomic nervous system and the hypophyseal-pituitary axis), the gut, microbiota, and the immune system influence intestinal functions such as motility, permeability, fluid secretion such as mucin, immune function like cytokine product modulation and microbial composition [26,29]. All of these factors are reported to be dysregulated in IBS [29]. Abnormalities seen in IBS include visceral hypersensitivity, central sensitization, intestinal dysmotility, alterations in the gut microbiota composition and structure, and chronic physiological stress [27,30].

The trillions of microbes residing in the gut are actively involved in the maintenance of normal physiology which includes energy metabolism by fermenting undigested carbohydrates and the development of the immune system of the host. [27,31]. The normal mucosa of the intestine consists of a layer of epithelial cells coated with mucus, which functions to entrap commensal microbes and secrete IgA. This secreted IgA prevents colony formation of pathogens along with other protease enzymes [30]. 

The vagus nerve functions as a major modulator in the gut-brain axis which is composed of somatic and afferent fibers and general and special visceral efferent fibers. It will modulate the integrity of the intestinal barrier, mucosal immune response, secretory functions, and motility to aid in the process of functional digestion [28,30]. The activation of the vagus nerve occurs as a result of gut microbes' response to different types of diets, metabolites such as short-chain fatty acids, enzymes, neurotransmitters such as serotonin, dopamine, acetylcholine, glutamate, γ-aminobutyric acid (GABA), and noradrenaline [30]. Thus, the changes caused by the vagus activity induce changes in the microbial habitat, resulting in abundant and high diversity of microbial taxa [28].

Diet is the most robust intervention to alter the gut microbiota [32]. A less diverse diet lacking essential nutrients will reduce the substrate availability for microbial growth, resulting in intestinal dysbiosis - a depletion of beneficial species and overgrowth of pathogenic species. Although it is hard to determine which specific dietary components adversely impact the gut microbiome, both long-term and short-term dietary changes impact the gut microbiota and their relationship with the host’s autonomic nervous system [4,27]. Chronic intake of carbohydrates, proteins, and animal fat is associated with increased Prevotella spp [27]. The Western diet rich in processed, fried, fatty, and sugary content is known to induce a reduction in microbial diversity. Dietary modifications such as low FODMAP and TDA have improved the symptoms and resulted in a change in the microbial composition [26,27,32]. Having a high saturated fatty acid (SFA) intake is associated with reducing the total bacterial community in humans and increasing the diversity of the microbial community [31]. Both Mediterranean and plant-based diets increase microbial diversity and promote healthy microbes. A diet rich in fats drastically impacts the gut microbes, resulting in a reduction in the Lactobacillus group and an increase in the Firmuculates, which results in increased permeability of the gut [26,33,34].

A high-fiber diet facilitates the growth of beneficial bacteria and reduces pathogenic bacteria [32]. Ingestion of fiber promotes hydrolytic bacteria growth thus increasing the diversity of the microbes in the gut [26]. Cellulose has been shown to change the composition of the microbes as well as influencing their enzymatic composition [32]. The short-chain fatty acids (butyric acid, propionic acid) are formed as a result of microbial processing of non-digestible dietary fibers, and they act on the endocrine cells to secrete certain chemicals such as Glucagon-like peptide-1 and Peptide-YY which return act on hypothalamic centers to control feeding behavior and energy balance [21,29,30 ]. These short-chain fatty acids (SCFAs) also act as the main energy source for colonocytes [32]. So, diets rich in fiber, phytochemicals, and live bacteria reduce inflammation and increase microbial diversity and production of SCFAs strengthening the gut barrier function [29,30]. 

Low FODMAP diet

The treatment approach for different subtypes of IBS should be tailored according to the specific symptoms experienced. In the case of IBS with predominantly diarrhea (IBS-D), the primary goal is to reduce excessive bowel movements. Conversely, for IBS with predominantly constipation (IBS-C), the focus is on promoting regular bowel movements. Individuals with IBS are generally advised to maintain a healthy diet by consuming smaller portions, avoiding foods that can produce gas or ferment in the gut, limiting alcohol and fat intake, as well as minimizing the consumption of spicy foods [34]. Despite the popularity of diets such as gluten-free and lactose-free diets among IBS patients seeking symptom relief, there is limited scientific evidence supporting their effectiveness unless the individual has specific conditions like lactose intolerance or celiac disease that require the elimination of these substances [35,36].

Several studies have provided evidence that the consumption of FODMAPs can elicit gastrointestinal symptoms [37-39]. Therefore, in recent years, there has been significant attention focused on a novel treatment option for IBS known as the low FODMAP diet. This dietary approach involves restricting the consumption of short-chain fermentable carbohydrates, including galacto- and fructo-oligosaccharides (GOS, FOS), lactose, fructose, and sorbitol. These carbohydrates have been identified as potential contributors to IBS symptoms [40].

FODMAP Hypothesis 

**Figure 3:**
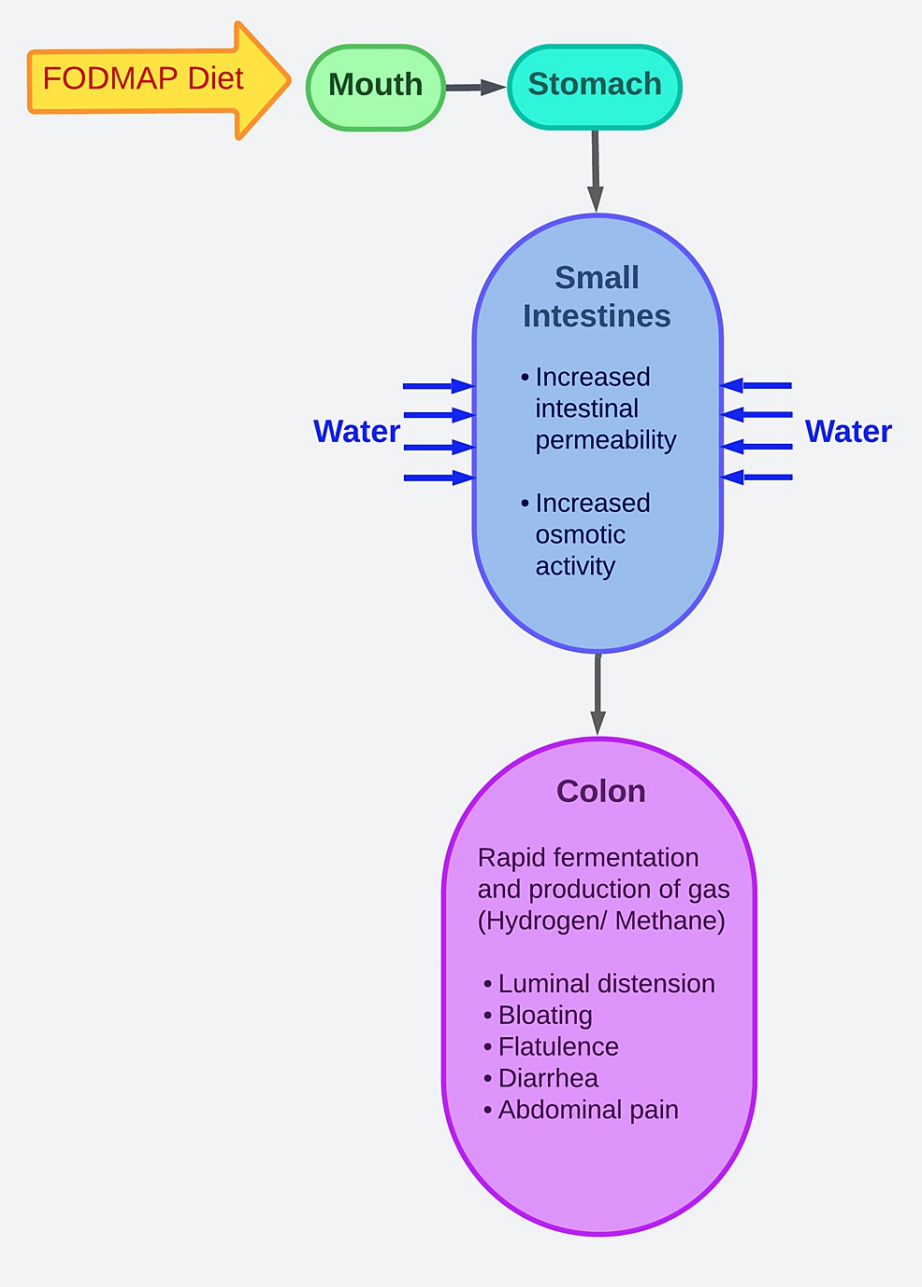
FODMAPs hypothesis FODMAP: fermentable oligosaccharides, disaccharides, monosaccharides and polyols This is an original artwork made by Dr. Dilushini Caldera.

Patients with IBS commonly have symptoms such as abdominal pain, bloating, excessive gas, and changes in bowel habits. Studies using barostats have revealed that these symptoms can be triggered by distension of the intestinal lumen [41]. It has been observed that FODMAPs can increase the total output of intestinal content, including the effluent water volume, thereby exerting osmotic activity. The excessive delivery and poor absorption of FODMAPs lead to their rapid fermentation and subsequent gas production in the colon. The occurrence of gas production from carbohydrate fermentation and fluid influx can result in distension of the lower small intestine and upper colon. In individuals with visceral hypersensitivity, these factors may contribute to symptoms such as bloating, excessive gas, and abdominal pain. The gut's response to distension can also cause changes in bowel habits, including constipation, diarrhea, or a combination of both. Moreover, increased fluid delivery to a healthy bowel can contribute to diarrhea, resembling the effects of an osmotic laxative [42,43]. Figure 3 is an illustration of the mechanism through which FODMAPs contribute to IBS symptoms.

Spectrum of FODMAPs

**Figure 4:**
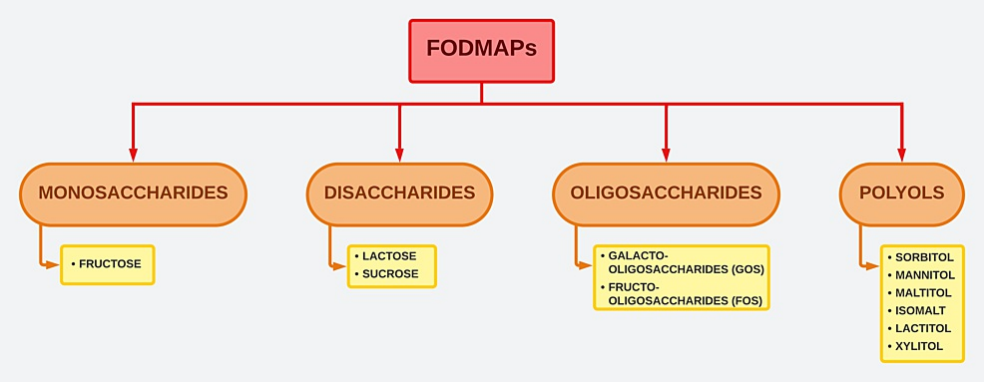
FODMAPs spectrum FODMAP: fermentable oligosaccharides, disaccharides, monosaccharides and polyols This is an original artwork made by Dr. Dilushini Caldera.

Figure 4 illustrates the spectrum of FODMAPs.

Fructose: Fructose is a naturally occurring component commonly found in fruits, berries, and various plants. It is also used as a dietary additive in confectionery, soft drinks, and diabetic food products. Fructose is present in various forms in the diet, including as a monosaccharide, as a component of sucrose (a disaccharide), or as polymerized fructans. When fructose is consumed together with glucose in equal amounts, such as in sucrose, it is absorbed with an efficiency estimated to be 85% of glucose. This absorption process is facilitated by glucose and has a high capacity. Additionally, in the presence of excess glucose, free fructose can be absorbed through a low-capacity, glucose-independent facilitated transport mechanism. While malabsorption may occur when a large amount of free fructose is present, it is important to note that around one-third of the population has a limited ability to absorb free fructose [38,44].

Fructo-oligosaccharides (FOS)/ Fructants: Fructans are chains of fructose molecules that can be linear or branched. They exist as β-2,1-linked inulins or β-2,6-linked levans. Inulin-type fructans are considered nondigestible oligosaccharides and have been extensively studied for their potential health benefits. While they possess nutritional value, they are also highly fermentable in the colon, which can lead to intestinal discomfort [45,46].

Galacto-oligosaccharides (GOS): GOS are oligosaccharides created through the process of β-galactosidase transgalactosylation. GOS is considered an indigestible component of food, as it can pass through the upper gastrointestinal tract with limited breakdown. However, higher quantities of galacto-oligosaccharides have been found to have adverse effects on individuals with gastrointestinal disorders. They are easily fermented in the colon, resulting in the production of excessive gas [47-49].

Lactose: Lactose is a commonly used ingredient in the production of sweets, confectionery, bread, and sausages. Lactose is broken down by an enzyme called lactase, which is predominantly present in the jejunum of the small intestine. When there is a deficiency of lactase, known as hypolactasia, it results in the malabsorption of lactose. This malabsorption can lead to symptoms of lactose intolerance, which may include loose stools, abdominal bloating, pain, flatulence, and nausea. The prevalence of lactase deficiency varies widely among different ethnic groups and countries [50].

Polyols: Among the commonly found polyols are sorbitol, mannitol, maltitol, isomalt, lactitol, and xylitol. Sorbitol can be naturally present in certain fruits and is also used as an ingredient in sugar-free sweet foods. Polyols are not well absorbed in the small intestine and are readily fermented in the gut [51-53].

Efficacy of Low FODMAP Diet in IBS 

Implementing a low-FODMAP diet in individuals with IBS, which involves restricting the intake of fermentable short-chain carbohydrates, proves to be an effective approach for managing IBS. However, this dietary therapy is associated with significant reductions in luminal bifidobacteria after four weeks. This decline is primarily attributed to the limited availability of fructans (including FOS) and GOS, which are important substrates for bacterial fermentation in the gastrointestinal tract. As a result, symptoms are reduced. The study subjects in most LFD studies are only followed up for a few weeks; therefore, the long-term effects of a low FODMAP diet have not been evaluated [54].

Several studies conducted on the effects of the low FODMAP diet in IBS patients have shown positive effects on the overall quality of life for these patients. Most of these studies have certain limitations, including a lack of specific information on the FODMAP content in the diet, resulting in high variability among control diets in meta-analyses. Additionally, the adherence of subjects to the diet has not been consistently assessed in some studies [55-58].

Low FODMAP-GFD

Current evidence suggests that multiple dietary components containing FODMAPs, gluten, wheat, lactose, and milk proteins act as triggers in the generation of symptoms in IBS [59,60]. Fructans, a type of FODMAP that is commonly included in foods with gluten was suggested to cause symptoms in patients with IBS in a recent randomized controlled trial, which studied the effects of gluten and fructans separately [61].

Gluten alters the total gut microbiota abundance which is attributed to the relative change in the Bifidobacteria cluster [59]. Clinically evident results suggest that using a low FODMAP- gluten-free diet improves the symptoms and quality of life in IBS patients, which can be attributed to the alteration of gut microbiota with significant levels of beneficial species [59,60]. But GFD is known to cause nutritional deficiencies, therefore it is important to identify the causative agent of IBS before implementing a restricted diet in patients with IBS [55,62].

A recent clinical trial study conducted by Naseri et al., including 30 IBS patients (Rome IV criteria), showed a significant improvement in the severity of symptoms and a reduction of fecal calprotectin (FC) level, owing to the regularization of gut microbial composition. This six-week study of a low FODMAP-gluten-free diet (LF-GFD) assessed the clinical symptoms using IBS symptom severity score (IBS-SSS), diversity of gut microbiota, and inflammation status by FC level, where the fecal samples were collected before and after the dietary change. Compared to the baseline, a notable reduction in IBS-SSS was seen following a LF-GFD along with a reduction in FC level. A large increase in Bacteroidetes was observed in the gut with a reduced Firmicutes to Bacteroidetes (F/B) ratio [59]. 

Symptoms of IBS often overlap with other disorders like non-celiac gluten sensitivity (NCGS). While LF-GFD is effective in improving IBS symptoms, a gluten challenge in the background of LF-GFD is used in the identification of NCGS in IBS patients [60,62].

LF-GFD is favorable in reducing anxiety and enhancing the quality of life in IBS patients. Fifty IBS patients followed an LF-GFD for six weeks followed by randomization into three groups as (a) gluten challenge with doubling the dose every two weeks (first week - 8g/day, second week - 16g/day, third week - 32g/day), or (b) adhere to the low FODMAP strict GFD or (c) gluten-containing diet without any restrictions. A dramatic improvement in symptom scores was observed at the end of the first six weeks with a reduction in pain severity, bloating, and the total symptom score in GFD and the gluten unrestricted group. Analysis between groups showed noticeable differences in pain severity, frequency, and quality of life [60].

LF-GFD followed by gluten/placebo challenge is a better method in the identification of NCGS in IBS patients but could be affected by FODMAP, particularly fructan, intolerance. Thirty IBS patients followed an LF-GFD for two weeks and the individuals showing a significant symptomatic improvement evaluated by a visual analog scale (VAS) were randomized to either gluten or placebo challenge. Following a dietary change with LF-GFD, 26 patients had a significant symptomatic improvement, and NCGS positivity was 46.1% based on VAS which had ≥30% increment, whereas this was only 19.2% with another method following a gluten challenge [62].

In a study performed by Nordin et al. to show the causative effect of FODMAPs and gluten on IBS symptoms, FODMAP showed a limited effect while gluten did not show any effect on IBS symptoms. This study randomized 103 participants (Rome IV criteria) to one of the intervention groups for one week, on a background of LF-GFD. Intervention groups were either FODMAP 50g/d, gluten 17.3g/d, or a placebo, with a week washout period between them and the analysis included patients who covered more than one intervention group. Significantly higher IBS-SSS was noted in the FODMAP group relative to the placebo and gluten groups while a similar IBS-SSS was observed between placebo and gluten groups [63]. Taken together, these findings suggest that the perceived benefit of a gluten-free diet in IBS may derive from the elimination not of gluten itself but of fructans via their FODMAP effects. 

Low FODMAP Diet and Probiotics

The low-FODMAP diet decreases the abundance of Bifidobacterium which could be altered favorably by supplementing with a Bifidobacterium-containing probiotic [59]. Probiotics are an easily accessible dietary option that benefits health when given in appropriate amounts [57,64]. In multiple studies, the effects of low FODMAP and probiotics on IBS symptoms, stool consistency and frequency, and gut microbial composition were assessed using various scoring symptoms, quantitative PCRs, volatile organic compounds (VOCs), and gene sequencing [57,64,65]. According to the pattern of VOCs in the fecal metabolome, IBS could be distinguished from other conditions making VOCs a potential diagnostic biomarker, affirming that an abnormal microbiome is associated with IBS [64].

LFD supplemented with a probiotic increased Bifidobacterium in fecal samples compared to the placebo along with a reduction in symptom severity scores in a study of 104 patients. Patients were randomized to LFD or a sham diet, supplemented with a multi-strain probiotic or a placebo for four weeks. Before and after the study symptomatic data and quality of life were assessed with standard scoring systems while RNA sequencing and quantitative PCR analyzed fecal samples. After the dietary intervention, 61% of LFD had significant symptom relief whereas it was only 39% in the sham diet, and similar relief was seen between patients given probiotics and placebo. Patients supplemented with probiotics had a higher level of Bifidobacterium in fecal samples compared to placebo, but LFD did not have any effect on the gut microbial environment [57].

Novel low-cost and non-invasive VOC profiling can assess the responsiveness of IBS patients to LFD and probiotics. Adult IBS patients (Rome III criteria) included in the 2×2 factorial study were randomly allocated to follow an LFD or a sham diet for four weeks, with each of them being randomly supplemented with either a multi-strain probiotic or a placebo, without interactions among them. A decrease of 50 or more points in IBS-SSS was the stated response, and VOCs were analyzed in fecal samples collected before and after the dietary intervention. In the end, 93 completed the study, with a response rate of 80% in LFD and 45% in sham diet, while probiotics and placebo had a similar response of 63% and 61%, respectively. Analyzed VOC showed a mean accuracy of 97% in response to LFD and 89% in probiotics [64].

Another study showed the buffering nature of probiotics on Bifidobacterium species, which is affected by LFD, by using a 2×2 factorial randomized controlled study published earlier involving 95 patients with IBS. Assessment of diet was done at four hierarchical levels and microbiota profiling used partial 16S rRNA gene sequencing. Multiple connections between diet and microbiota were observed at the dietary level, with a negative connection between protein and Bifidobacterium quantity. Alterations in the quantity of major saccharolytic genera were found following an LFD compared with a sham diet, with higher levels of Bacteroides and low Bifidobacterium. Supplementation with probiotics elevated the abundance of Lactobacillus and Streptococcus compared to the placebo, thus mitigating the impact of low FODMAP on Bifidobacterium [65]. 

Two randomized controlled trials assessed the effectiveness of low FODMAP in combination with a prebiotic containing β-Galactooligosaccharide (B-GOS) and a probiotic containing *Bacillus coagulans* respectively, on IBS symptoms and fecal Bifidobacteria [66,67].

Greater symptomatic improvement was produced in the LFD/B-GOS combination compared to the sham diet/placebo, despite the reduction of bifidobacteria with 1.4g/d B-GOS and in long-term LFD-affected fecal Actinobacteria and butyrate. The three-arm trial recruited 69 IBS patients (Rome III criteria) for four weeks, and the patients were randomly allocated to either a sham diet supplemented with a placebo (control) or LFD with either a placebo or 1.4g/d B-GOS. Before and after four weeks, gastrointestinal symptoms were evaluated along with fecal microbiota, fecal short-chain fatty acids, pH, and urine metabolites. After four weeks, symptomatic improvement was higher at 67% in the LFD/B-GOS group compared to 30% in the control group. LFD and LFD/B-GOS had similar Bifidobacterium concentrations, but LFD/B-GOS had a lower concentration compared to the control group. The control group showed a higher Actinobacteria proportion compared to the LFD/B-GOS and LFD, along with a higher fecal butyrate level than LFD and LFD/B-GOS [66]. 

LFD with probiotic *Bacillus coagulans* showed superiority to LFD alone in improving IBS symptoms. Fifty IBS patients (Rome IV criteria) were randomized to LFD supplemented with either a probiotic containing 109 *Bacillus coagulans* spores and 400mg inulin or a placebo containing 500mg rice starch for eight weeks. Both groups experienced significant improvements in defecation consistency, quality of life, abdominal pain severity and frequency, abdominal distension, and self-reported severity score. However, in the LFD/probiotic group, more frequent patients had a significant improvement in the IBS-symptom severity score than the LFD/placebo group [67].

Mediterranean diet

The Mediterranean diet (MedDiet) is a diet inspired by countries bordering the Mediterranean Sea [68]. MedDiet includes the regular consumption of olive oil, fresh fruits, vegetables, whole grains, legumes, and nuts. It also includes moderate consumption of fish, white meat, eggs, and fermented dairy products (e.g.: yogurt and cheese) and rare sweets and red meat. Water is used as the primary beverage, with red wine accompanied by meals in moderation [69,70]. A hallmark of the MedDiet is that it is composed of unprocessed, fresh foods that are abundant in nutrients, in contrast to the Western diet which is rich in processed and ultra-processed foods that lack nutrients [70]. The MedDiet has a high fat intake, accounting for 40%-50% of total daily calories of which 15%-25% are monounsaturated fatty acids mainly derived from olive oil, in contrast to saturated fatty acids that account for less than 8% of the consumed daily fat.

Various studies have shown that adherence to the MedDiet results in lower concentrations of inflammatory markers. The ATTICA study showed a 20% decrease in CRP and a 17% decrease in interleukin-6 (IL-6) after following a MedDiet [69]. MedDiet is characterized by a high omega-3 fatty acid intake obtained from fish, nuts and seeds, plant oils, and fortified foods and it has a low Omega-6:Omega-3 ratio (2:1-1:1) when compared to Western diets (14:1) [68,70]. Low Omega-6:Omega-3 ratio is known to reduce inflammation [71]. The abundant fiber in the MedDiet is associated with improved barrier integrity and motility, and reduced inflammation [69]. The abundant consumption of dietary fiber, and anti-inflammatory and antioxidant compounds may act synchronously to produce beneficial health outcomes [70].

Although the low-FODMAP (LFD) diet is known to alleviate IBS symptoms, its ability to reduce inflammation, which is one of the cardinal mechanisms contributing to symptom development in IBS, has not been proven yet. On the other hand, MedDiet has antiinflammatory benefits owing to olive oil (which contains around 30 phenolic compounds) and diverse fibers, inducing immunomodulating properties that increase the amount of beneficial bacterial species in the intestine [69].

A prospective, cross-sectional, case-controlled study involving 100 IBS patients, aged 12-18 years, investigated the effects of adherence to the MedDiet on IBS using IBS scores (Irritable Bowel Syndrome Severity Scoring System (IBS-SSS), Irritable Bowel Syndrome Quality of Life (IBS-QoL), and total score) and clinical and laboratory parameters. The study divided the children into two groups, one receiving a regular diet and the other given MedDiet. The study found that the MedDiet was safe and well-tolerated by the patients. Adherence to MedDiet was associated with improvement in the IBS-SSS (from 237.2 to 163.2), IBS-QoL (from 57.3 to 72.4), and total IBS score (from 24.1 to 28.8) when compared to the group that received a regular diet that showed no significant improvement in the scores [72].

Another study conducted by Zito et al. evaluated the relationship between low adherence to MedDiet and the presence of IBS and functional dyspepsia (FD). A total of 1134 subjects were recruited and investigated based on their dietary habits and the presence of functional gastrointestinal symptoms. It was found that low adherence to MedDiet may result in functional gastrointestinal symptoms, which was demonstrated by lower adherence scores in females with FD and IBS (0.58 and 0.22 respectively). Age categories 17-24 and 24-34 with IBS also showed a low adherence score of 0.45 and 0.44 respectively. However, this study was performed in one region [73].

A study done by Chen et al. studied the link between Mediterranean diet and IBS symptoms. The study involved 106 IBS patients and 108 health control (HC) participants. All participants were given questionnaires to complete about diet history and gastrointestinal symptoms. Adherence to MedDiet was determined using the Alternate Mediterranean Diet (aMED) and Mediterranean Diet Adherence Screener (MEDAS) and food items that cause IBS symptoms were identified using sparse partial least square analysis. Stool samples were also collected for 16S ribosomal RNA gene sequencing to assess the microbial composition in IBS patients. It was found that there was no difference in the aMED and MEDAS scores between IBS and HCs and the scores did not correlate with IBS-SSS, abdominal pain, or bloating. Consuming greater amounts of fruits, vegetables, sugar, and butter were linked to an increase in IBS symptoms and the study also found that certain MedDiet foods were also linked to an increase in IBS symptoms. Adherence to symptom-modified MedDiet showed a decrease in harmful bacteria such as Faecalitalea, Streptococcus, and Intestinibacter and an increase in beneficial bacteria such as Holdemanella from the Firmicutes phylum. The study conducted was a cross-sectional study; therefore, the participants were not randomised into specific dietary interventions, and the results only demonstrated the association and not the cause. Also, diet was assessed using the Diet History Questionnaire II (DHQ II) which depends on recalling diets in the past year and does not involve lifestyle components of the MedDiet. In addition to this, the study population was based in Los Angeles which resulted in a below-average MedDiet score when compared to the Mediterranean population; also, the study population consumed less olive oil when compared to MedDiet studies [74].

Tritordeum based diet

It is well known that people with IBS experience worsening symptoms after consumption of foods high in FODMAPs. Foods such as wheat and beans contain these short-chain carbohydrates and, therefore, may cause aggravation of GI symptoms due to poor absorption of it by the small intestine. LFDs have shown promising results in terms of alleviating the symptoms and reducing inflammation [75]. However, compliance with LFD is problematic as it requires constant advice from a dietician and restriction of certain foods [75,76]. In addition, 50% of IBS patients did not show improvement in their symptoms after following an LFD [77]. Thus, new dietary options based on less immunogenic cereals, such as the tritordeum-based diet (TBD) have recently gained interest in the management of IBS [75,76].

Tritordeum is a cereal derived from the crossing of durum wheat and wild barley. It is grown in Spain, Portugal, and Apulia [78]. Cultivation of this cereal does not need much care and is resistant to adverse conditions like drought, heat, and disease [77,78]. TBD has the unique characteristic of having lower levels of gliadin, reduced immunogenic gluten peptide concentrations, and fewer carbohydrates and fructans than bread wheat [76,77]. This grain also has a high content of protein, fiber, phenolic compounds, and natural antioxidants such as lutein [76].

TBD is not suitable for patients with celiac disease due to the presence of gluten, but it is suitable for patients with non-celiac wheat sensitivity (NCWS), IBS-D, or IBS-M as symptoms like abdominal bloating may reduce due to its low gluten and fructans content [75,77]. The reduced content of gliadin, fructans, and carbohydrates in TBD has resulted in the alteration of several pathophysiological mechanisms such as alteration of intestinal permeability, dysbiosis, and pro-inflammatory immunological profile [78].

Russo et al. conducted a pilot study to evaluate the effects of adhering to a 12-week TBD on the GI symptoms (assessed by a questionnaire) and the integrity of the GI barrier (evaluated by sugar absorption test, markers of integrity, and functions) in 16 patients suffering from IBS-D. It was found that TBD reduced the IBS-D symptoms by reducing the intestinal permeability, decreasing levels of markers of integrity, decreasing mucosal inflammation and fermentative dysbiosis, thereby, improving the GI barrier. The limitations of the study are that the cohort is too small to draw strong conclusions and the finding of fermentative dysbiosis was not supported by data since the bacterial population of the GI tract was not analyzed. More research is needed to understand the pathophysiological mechanisms linking wheat consumption and the IBS profile [77].

Another study that evaluated the effects of adhering to TBD for 12 weeks on GI symptoms, anthropometric and bioelectrical impedance parameters, and psychological profiles in 18 female patients suffering from IBS-D with abdominal bloating as the main symptom found that the IBS-SSS “Intensity of abdominal bloating” was reduced with an improvement in the anthropometric profile. An altered psychological profile was also observed with a reduction in anxiety, depression, somatization, interpersonal sensitivity, and phobic and avoidance manifestations after TBD and a correlation between anxiety and the intensity of abdominal bloating was also reported. However, there was no correlation between “Intensity of abdominal bloating” and “Abdominal circumference”. The limitations of the study are that the research was not a double-blind controlled design and there was no control group, which would have been beneficial in evaluating subjective responses. Also, the anthropometric and BIA parameters evaluated in this study were not influenced by a placebo and are in line with each other, the symptoms, and the previous study they conducted. Lastly, the study did not investigate whether the psychological factors precede abdominal symptoms as it would require a large number of patients [78]. A similar study that compared the effects of 12 weeks of LFD with 12 weeks of TBD showed that both diets improved IBS-D symptoms and QoL, which was demonstrated by a reduction in the IBS-SSS score. Also both the dies did not alter the micronutrient content [75].

Another study by Caponio et al. which studied the changes in the fecal metabolome composition in two groups subjected to 12 weeks of TBD and LFD found that there were significant changes in the fecal volatile organic compounds (VOCs) in both groups with increased decanoic acid in the TBD group and increased nonanal and ethanol in the LFD group. Also, SCFAs, which are known to cause inflammation, were found to be reduced in both groups. This study had a few limitations in terms of a small cohort size and short duration of study. Long-term studies with a large population will be able to evaluate the effect of long-term adherence to these diets on the fecal metabolome. Also, it would be useful to do a 16S meta-taxonomic analysis of the bacterial populations to describe the changes observed in the fecal metabolome [76]. Overall, all these studies suggest that TBD is associated with improvement in IBS-D symptoms and QoL [75-78].

Traditional dietary approaches and low FODMAP diet clinical trials 

Traditional dietary approaches are still chosen to be the most favorable option in non-constipated IBS patients. Patients in Rome IV criteria in non-constipated IBS were randomized into three dietary approaches: TDA (Traditional dietary approach), LFD (low FODMAP diet), and GFD (gluten-free diet). They were assessed about their quality of life with the dietary therapy, changes in nutritional intake, and stool dysbiosis alterations. Although the LFD diet had the lowest FODMAP content, the macro and micronutrients have not changed significantly among the three diets. The stool dysbiosis index was similar across the diet. However, TDA was found to be easier to follow among the responders because it’s cheaper and less time-consuming [79].

The LFD (Low FODMAP diet) and TDA (Traditional dietary approach) both have reduced symptoms in Chinese IBS-D patients however, symptomatic improvements were achieved much earlier in the LFD diet with regards to symptom improvement in the frequency of stool passing and excessive wind. One hundred and eight Chinese IBS-D patients (Rome III criteria) were randomized to an LFD or TDA diet where their fecal samples were collected before and after the diet change. The stools were assessed for changes in the SCFAs and the microbiota presence. The presence of less carbohydrate-fermenting bacteria resulted in decreased saccharolytic fermentation activity thus improving symptoms of the patients in LFD [80]. The findings of observational and experimental trials using various dietary treatments for the management of IBS symptoms are summarised in Table 1.

**Table 2:** A summary of observational and experimental studies describing various dietary treatments for the management of IBS symptoms IBS: irritable bowel syndrome; FODMAP: fermentable oligosaccharides, disaccharides, monosaccharides and polyols; IBS-SSS: IBS symptom severity scale; IBS-D: irritable bowel syndrome with diarrhea; FC: fecal calprotectin; GFD: gluten-free diet; NCGS: non-celiac gluten sensitivity; IBS-M: irritable bowel syndrome mixed type; IBS-U: irritable bowel syndrome-unsubtyped; IBS-C: irritable bowel syndrome with constipation; LFD/B-GOS: low fodmap diet/ β-Galactooligosaccharide; LFD: low fodmap diet; aMED: alternate mediterranean diet; MEDAS: mediterranean diet adherence screener; HC: health control; DHQ II: diet history questionnaire II; MD: mediterranean diet; TBD: tritordeum based diet; BIA: bioelectrical impedance analysis; QoL: quality of life; SCFAs: short-chain fatty acids; CNAQ: council of nutrition appetite questionnaire

Author	Type of Study	Sample Size	Inclusion criteria	Results of study	Study weakness
Naseri et al. [59]	Uncontrolled, open-label clinical trial study	30	A diagnosis of IBS according to Rome IV criteria, age 18- 59, absence of other functional gastrointestinal (GI) diseases, normal mucosa on biopsy and colonoscopy. Exclusion criteria: positive history of inflammatory bowel disease, celiac disease, diseases of the liver, GI surgery, cancer, use of non-steroidal anti-inflammatory drugs (NSAIDs), use of alcohol, use of immunosuppressive agents systemically, poorly controlled psychiatric diseases, presence of fissures, hemorrhoids, and microscopic colitis on biopsy specimens and colonoscopy. Usage of any drugs that affect bowel function four weeks before the study, such as broad-spectrum antibiotics, and probiotics.	Reduction in IBS-SSS in 73.3% of patients with 3.3% having >60% and 53.3% having 30-60% reduction, increased level of unfavorable gut microbiota Bacteroidetes (11.69%- 26.65%), reduction in Firmicutes (31.59%- 22.17%), increased relative abundance of Bifidobacterium and Lactobacillus at the genus level, significant reduction in Firmicutes to Bacteroidetes (F/B) ratio (2.6:1 – 0.8:1), reduction in the level of FC	Small sample size and patients predominantly being IBS-D
Saadati et al. [60]	Randomized single-blinded controlled clinical trial	50	Adults who meet Rome IV criteria, aged 18- 80 years. Exclusion criteria: the presence of psychological disorders, major abdominal surgery, diabetes mellitus, pregnancy, making dietary changes during the study, unwillingness to continue, following a GFD or low FODMAP diet in the past six months,	Significant reduction in pain severity and frequency. Significant reduction in the anxiety levels. Improvement of the quality of life.	Small number of patients, limited follow-up time
Barone et al. [62]	Randomized double-blind placebo-controlled crossover trial	30	A diagnosis of IBS according to Rome IV criteria, no serious symptoms, no use of any drugs for the abnormalities in bowel habits in the previous three months. Exclusion criteria: Use of a GFD in the previous six months, presence of any of the following diseases; celiac disease or wheat allergy, chronic intestinal inflammatory diseases, major abdominal surgery, psychiatric problems, diabetes mellitus, pregnancy or prior anaphylactic episodes	Significant improvement in the symptoms following a low FODMAP-GFD	Limited number of patients in the sample, difficulty to exclude patients with seronegative celiac disease among NCGS patients, use of Di Sabatino criteria in the evaluation which is not routinely applicable
Nordin et al. [63]	Double-blind placebo-controlled, randomized three-way crossover trial	103	Fulfilling IBS Rome IV criteria with moderate to severe IBS, BMI 18.5-38kg/m^2^, age18-70 years, hemoglobin 120-160g/L, transglutaminase immunoglobulin A <7 U/ml, C-reactive protein <5 mg/L, thyroid stimulating hormone <4 mU/L, systolic/ diastolic blood pressure ≤160/≤105 mmHg	Higher IBS-SSS in FODMAP group in comparison to the gluten and placebo groups, higher frequency of abdominal pain during FODMAPs intake	Exposures limited to seven days, low impact diets were provided as dietary advice instead of ready-made meals, adherence to the diet was assessed based on self-reporting
Staudacher et al. [57]	2×2 factorial, multicenter, randomized, placebo-controlled trial	104	IBS-D, IBS-M, IBS-U patients fulfilling Rome III criteria. IBS-C excluded. Age 18-65 years	Significant symptom relief following LFD (61%), a significant increase in the Bifidobacterium in fecal samples after supplementation with probiotic	Maintenance of blinding is difficult, excluded constipation-predominant IBS patients, difficulty to identify which dietary item (collective FODMAPs or one or individual FODMAP) cause the response, problem of collinearity, dietary changes might impact indirectly on microbiota composition by changing other physiological parameters like transit time, raised questions regarding usage of a dichotomous endpoint as a primary outcome
Rossi et al. [64]	2×2 factorial, multicenter, randomized, placebo-controlled trial	93	Fulfilling Rome III criteria, without any other significant medical problems	Significant change in IBS-SSS in LFD (80%) compared to Sham diet (45%), Similar response rate for both probiotic (63%) and placebo (61%)	A novel study without data prior data, need for a larger cohort to determine the validity of the study, limited information about the mechanisms linked to VOCs since it only determines VOC patterns, results could be unclear due to 2×2 factorial design of the study
Staudacher et al. [65]	2×2 factorial, blinded, randomized placebo-controlled trial	95	Age 18-65 years, diagnosed with IBS based on Rome III criteria	Increased abundance of Lactobacillus and Streptococcus with probiotic supplementation, mitigation of the effect of low FODMAP on Bifidobacterium	Collection of dietary data using food records, cut off for inclusion of operational taxonomic units for analysis together with adjustments for multiple comparisons could result in a type 2 error and mask true diet-microbiota relationships
Wilson et al. [66]	Randomized placebo-controlled three-arm trial	69	Fulfilling Rome III criteria, age 18-65 years	Significant symptom relief of 67% in LFD/B-GOS group, lowering of Bifidobacterium concentration, actinobacteria proportion and fecal butyrate in LFD/B-GOS group	Unclear effect of prebiotic, low dose of prebiotic in the LFD/B-GOS group to increase the Bifidobacteria
Abhari et al. [67]	Randomized controlled trial	50	Fulfilling Rome IV criteria	Significant improvement in IBS-SSS in LFD/ Bacillus coagulans group, higher frequency of patients with improved IBS-SSS in the same group	Presence of inulin in probiotic capsules which is a FODMAP
Al-Biltagi et al. [72].	Prospective cross-sectional case-controlled study	100	Children and adolescents diagnosed with IBS according to the Rome IV criteria, aged between 12-18 years	Mediterranean diet was found to be safe and well-tolerated among IBS patients. In the group following the Mediterranean diet, the IBS-SSS score decreased from 237.2 ± 65 to 163.2 ± 33.8, Mean IBS-QoL improved from 57.3 ± 12.9 to 72.4 ± 11.2 and the mean total IBS score increased from 24.1 ± 10.4 to 28.8 ± 11.2 by the end of the study. However, no significant improvement was seen in the group following the regular diet.	It is a cross-sectional study therefore it is unable to conclude the causality and the same was from a single center therefore cannot generalize the study results
Zito et al. [73].	Prospective study	1134	Age between 17-83 years	The study found significantly low adherence to a Mediterranean diet in the groups with IBS and FD symptoms (0.57 ± 0.23, and 0.56 ± 0.24 respectively) when compared to the control group (0.62 ± 0.21). Females had significantly low adherence scores to a Mediterranean diet in both IBS and FD groups, while for males it was only significantly low in the FD group. The study also showed that the adherence score was significantly low in the age groups 17-24 and 25-34 for FD and IBS when compared to the control group.	The study was performed only in one region
Chen et al. [74].	Retrospective cross-sectional study	214	IBS patients diagnosed according to Rome III/IV criteria and health control participants	"There was no difference in the aMED and MEDAS scores between IBS and HCs. Standard MD did not increase IBS symptoms although certain MD foods were associated with an increase in IBS symptoms. Adherence to a symptom-modified MD diet showed a decrease in harmful bacteria such as Faecalitalea, Streptococcus, and Intestinibacter and an increase in beneficial bacteria such as Holdemanella from the Firmicutes phylum suggesting that a personalized MD diet may benefit patients with increased IBS symptoms.	Participants were not randomized into specific dietary interventions and the results only demonstrated the association and not the cause. The DHQ II which assessed diet was based on recalling diet in the past year and did not involve lifestyle components of the MD. The study was based in Los Angeles which resulted in a below-average MD score when compared to the Mediterranean population, and the study population consumed less olive oil when compared to MD studies.
Russo et al. [77].	Pilot study	16	Patients diagnosed with IBS-D according to the Rome IV criteria aged between 18-65 years.	TBD reduces the IBS-D symptoms by reducing intestinal permeability, decreasing levels of markers of integrity, decreasing mucosal inflammation, and fermentative dysbiosis.	The cohort is too small to draw strong conclusions and the finding of fermentative dysbiosis was not supported by data since the bacterial population of the GI tract was not analyzed.
Riezzo et al. [78].	Prospective cohort study	18	Patients with IBS-D according to Rome III/IV criteria between ages 18-65	The IBS-SSS “Intensity of abdominal bloating” was reduced with an improvement in the anthropometric profile. An altered psychological profile was also seen with a reduction after TBD. A correlation between the intensity of abdominal pain and anxiety was also reported. However, there was no correlation between “Intensity of abdominal bloating” and “Abdominal circumference”.	The study was not a double-blind controlled design and there was no control group to evaluate the subjective responses. The anthropometric and BIA parameters evaluated in this study were not influenced by a placebo and are in line with each other, the symptoms, and the previous study they conducted. The study did not investigate whether the psychological factors precede abdominal symptoms.
Russo et al. [75].	Randomized-controlled trial	72	Patients with IBS-D according to Rome III/IV criteria	Both TBD and LFD improved GI symptoms and QoL, which was demonstrated by a reduction in the IBS-SSS. However, both diets did not alter the micronutrient content during the study period.	
Caponio et al. [76].	Randomized-controlled trial	38	Patients with IBS-D according to Rome IV criteria	Significant changes in the fecal volatile organic compounds (VOCs) were seen with increased levels of decanoic acid in the TBD group and increased ethanol and nonanal in the LFD group. SCFAs were found to be reduced in both groups.	Small cohort size and short duration of study.
Rej et al. [79]	Randomised controlled trial	114 (TDA ¼ 35, LFD ¼ 33, GFD ¼ 33)	Adults aged 18 years with Rome IV IBS-D or IBS-M, and an IBS-SSS of >75. Additional inclusion criteria included being English literate, able to travel to the hospital, and having telephone or internet access	The study group concluded that TDA was cheaper & easier to follow. The individuals who followed the LFD diet had a significant improvement in depression than those who followed the TDA diet. The individuals who followed LFD also had a significant improvement in dysphoria compared with TDA and GFD. However, changes in anxiety, somatization, and IBS QoL did not differ across all three groups.	The food frequency questionnaire tool used (CNAQ) was based on the Australian diet.
Zhang et al. [80]	Randomised controlled trial	108	Adult patients meeting Rome III criteria for IBS-D who had no abnormal results in blood or stool tests with a normal colonoscopy within the prior 2 years.	In the LFD group, FODMAP intake was reduced to a similar extent in responders and non-responders, whereas FODMAP intake in the TDA group remained at baseline amounts.	

## Conclusions

Irritable bowel syndrome (IBS) is one of the most typically diagnosed gastrointestinal disorders that remarkably affects patients’ lives. Even now, IBS treatment is challenging and requires a complete explanation of the disease. Dietary advice is a matter of utmost importance in the treatment of this disease. To improve the symptoms, elimination diets, including low FODMAPs and gluten-free diets, and traditional dietary advice like eating small regular meals, cutting back on alcohol and caffeine, and avoiding trigger foods worth mentioning. This article summarizes the effects of different types of diet on IBS and its symptoms.
